# Validation of Gender Identification From Frontal Sinuses Using Yoshino's Classification: A Morphometric Radiographic Study

**DOI:** 10.7759/cureus.59895

**Published:** 2024-05-08

**Authors:** Hanmandla Rajini, Raj Kumar Badam, Nallan Csk Chaitanya, Komali Garlapati, Divya Harika Pedada, Ayesha Aiman, Poojitha Gone, Sri Sahithya Kataram

**Affiliations:** 1 Oral and Maxillofacial Radiology, Panineeya Institute of Dental Sciences & Research Centre, Hyderabad, IND; 2 Oral Medicine and Radiology, Panineeya Institute of Dental Sciences & Research Centre, Hyderabad, IND; 3 Oral Medicine, Panineeya Institute of Dental Sciences & Research Centre, Hyderabad, IND

**Keywords:** discriminative function analysis, forensic dentistry, frontal sinus, gender determination, posteroanterior view

## Abstract

Introduction: Gender determination of deceased individuals may become necessary in scenarios involving sudden and unforeseen fatalities like explosions, fires, transportation accidents, or instances of mutilation or decomposition, which frequently require medicolegal expertise. Forensic radiology is instrumental in identifying gender. The shape of the frontal sinus is considered distinct for every person, differing even among identical twins, much like individual fingerprints and establishing personal identity.

Aims and objectives: This study was designed to validate and determine gender identification by evaluating frontal sinus measurements using digital posteroanterior cephalograms with reference to Yoshino's classification and to determine gender based on measurements of frontal sinus size, bilateral asymmetry, the outline of the upper border of the frontal sinus, partial septa, and supraorbital cells of the frontal sinus.

Materials and methods: A total of 300 digital posteroanterior cephalograms (150 males and 150 females) of age groups ranging from 18 to 30 years were collected from the records of the Department of Oral Medicine and Radiology, Panineeya Institute of Dental Sciences & Research Centre, Hyderabad. The parameters that were assessed in a digital radiograph are Yoshino's frontal sinus pattern of the individual, which includes frontal sinus size, bilateral asymmetry, superiority of the side, outline of the upper border, partial septa, and supraorbital cells. The measurements were taken, tabulated, and compared with the standard values of the gender measurement. The values were subjected to statistical analysis.

Results: Results showed a statistically significant difference in the mean height (p=0.000), width (p=0.000), area (p=0.000), partial septa (p=0.002), and outline of the upper border on the right side (p=0.011) of the frontal sinus for both males and females. The mean values for length, width, and area of the frontal sinus were higher in males than females. No statistical difference is found on the outline of the upper border on the left side, superiority of the side, and supraorbital cells. The application of discriminative analysis to the data accurately identified gender in 65.3% of the cases.

Conclusion: Thus, from the above study, the forensic application of frontal sinus morphology can be recommended as an adjunctive tool for gender determination.

## Introduction

Gender identification is crucial in forensics for victim and witness recognition, criminal profiling, and understanding crime dynamics. In sudden and unexpected fatalities, like explosions, fires, accidents, or criminal incidents, determining the deceased person's gender requires specialized medicolegal expertise [1]. Gender identification is crucial in legal matters, including insurance claims, will administration, business transactions, spouse remarriage, and negligence lawsuits [2]. Various methods, including bone analysis, have been employed for such identification, with the pelvis and skull emerging as pivotal in gender determination [3,4].

While the pelvis and skull are reliable indicators, the latter may not always be fully recoverable, prompting the exploration of alternative structures like the frontal sinuses [4]. These intricately shaped and individually distinctive lobulated cavities have drawn attention to their potential in post-mortem identification since Schuller's pioneering study in 1921 [5]. The frontal sinus, developing during childhood and maturing by age 20, undergoes unique changes influenced by age, growth spurts, and gender-specific patterns. Its irregularities, shaped by factors like age and trauma, render it an intriguing marker for individual identification [6].

The intricate anatomy of the facial skeleton has led to the creation of various radiographic methods, with one frequently employed approach being the posteroanterior (PA) view of the skull. This technique is utilized explicitly in the article to assess the frontal sinus. This research delves into the validation of gender identification through a morphometric radiographic approach, specifically employing Yoshino's classification [7]. By scrutinizing the intricacies of frontal sinus patterns, this study aims to contribute valuable insights to the field, paving the way for more reliable methods in forensic anthropology and aiding in determining gender with enhanced precision.

Objectives

The study has three key objectives: (1) determining gender through frontal sinus size measurements, (2) identifying gender based on bilateral frontal sinus asymmetry, and (3) assessing gender through the outline of the upper border and features like partial septa and supraorbital cells in the frontal sinus. These varied criteria aim to comprehensively understand gender determination based on frontal sinus morphology.

## Materials and methods

Study design

This study employs Yoshino's classification to determine gender from frontal sinus features, analyzing 300 digital posteroanterior cephalograms of individuals aged 18-30. Ethical approval was obtained from the Institutional Ethics Committee (IEC) of the Panineeya Institute of Dental Sciences & Research Centre (approval number: PMVIDS & RC/IEC/OMR/DN/354-20).

Sample

Radiographs meeting the inclusion criteria were collected from the Department of Oral Medicine and Radiology, Panineeya Institute of Dental Sciences & Research Centre. The study group comprised 300 digital PA cephalograms of individuals aged 18-30. The inclusion criteria were as follows: (1) age between 18 and 30 years, any gender, attending the Department of Oral Medicine and Radiology and (2) only high-quality radiographs with no visible errors. In contrast, the exclusion criteria were the following: (1) individuals with a history of orthodontic treatment or orthognathic surgery, (2) individuals with a history of trauma or any surgery of the skull, (3) individuals with any systemic disturbance affecting growth, and (4) individuals with hereditary facial and congenital anomalies.

Methodology

**Figure 1:**
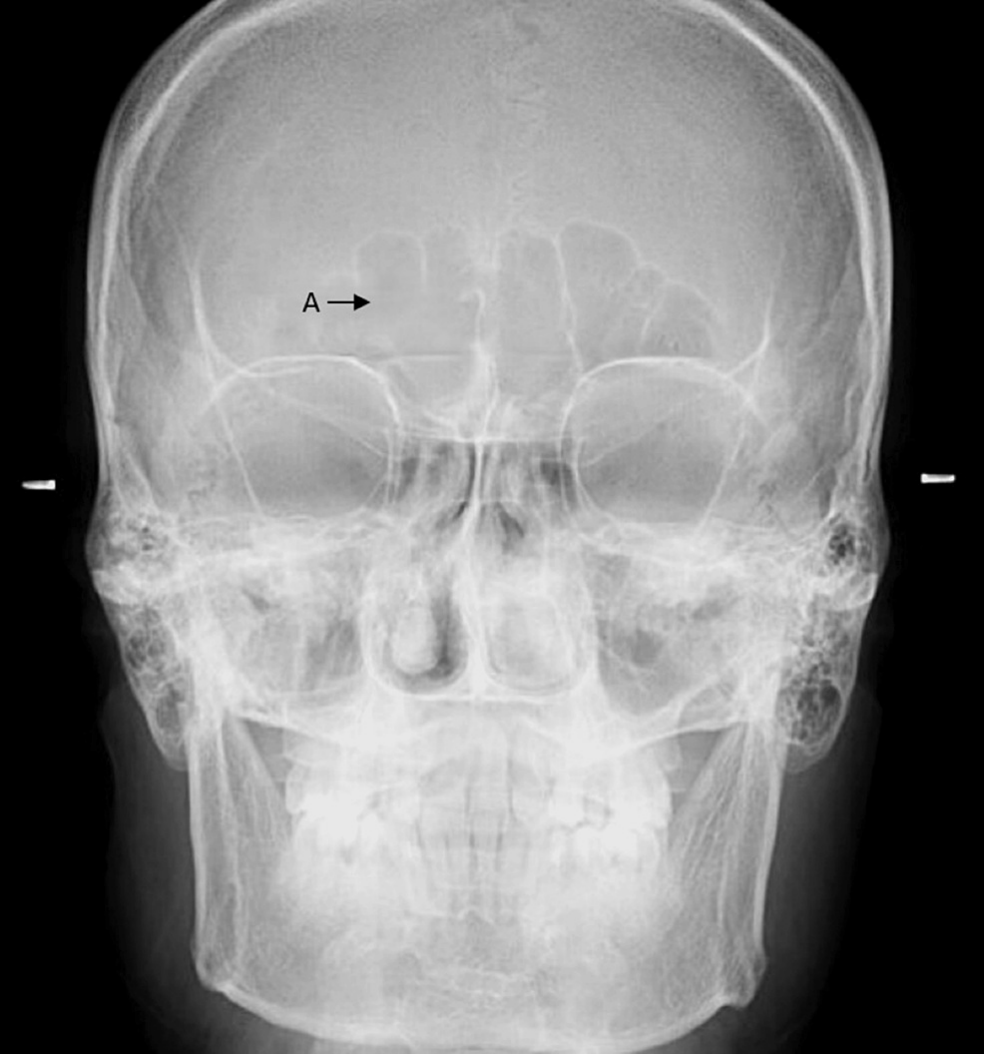
Assessment of frontal sinus parameters using a PA cephalogram "A" indicates frontal sinus PA: posteroanterior

For this study, 300 digital PA cephalograms representing individuals aged 18-30 were selected through simple random sampling. The radiographic images were saved as high-resolution JPEG files and imported to Adobe Photoshop CS3 extended for the assessment of frontal sinus parameters (Figure 1), including size, bilateral asymmetry, side superiority, upper border outline, partial septa, and supraorbital cells following Yoshino's classification. Measurements of frontal sinus size encompassing height, width, and area were performed by determining the maximum distance between the lowest and highest extents for height and the medial and lateral extents for width on both sides. The resulting linear measurements were expressed in centimeters, while areas were represented in square centimeters.

Statistical analysis

The obtained data was measured, tabulated on an Excel sheet, and subjected to statistical analysis using IBM SPSS Statistics for Windows, Version 20.0 (Released 2011; IBM Corp., Armonk, New York, United States). The Mann-Whitney U test, a non-parametric alternative to the independent sample t-test, was employed to compare means from the same population and determine equality. The chi-squared test for independence, also known as Pearson's chi-squared test, was utilized to investigate relationships between two categorical variables.

## Results

**Figure 2:**
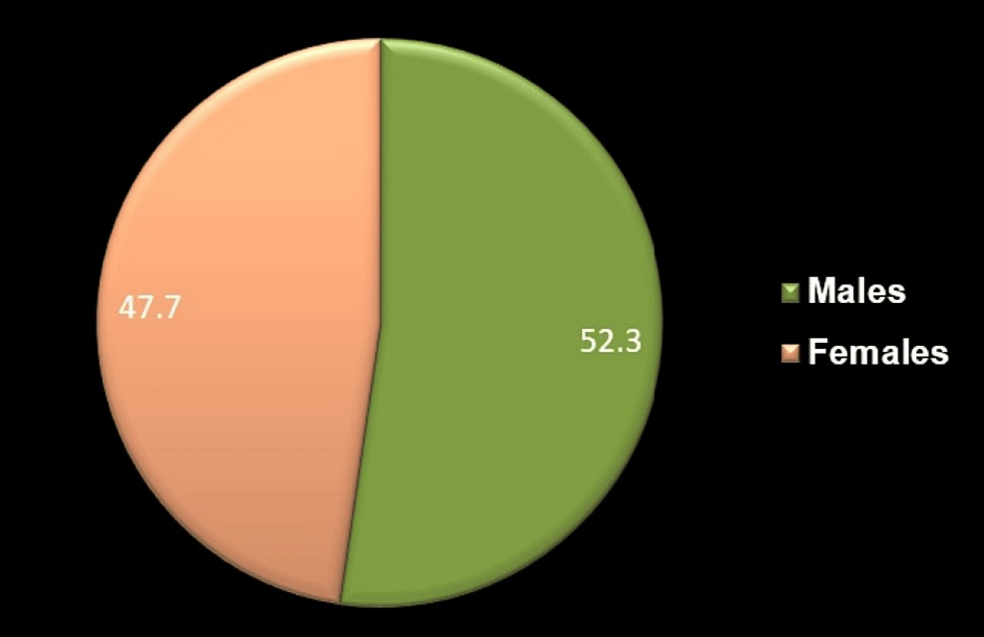
Demographic analysis

The study aimed to determine gender through frontal sinus measurements using digital PA cephalograms following Yoshino's classification. Initially, 300 subjects (150 males and 150 females) were included, but 13 males and 25 females were excluded due to aplasia. Consequently, the final sample comprised 137 males (52.3%) and 125 females (47.7%) (Figure 2). Bilateral absence of the frontal sinus was observed in 12 cases (4%), with seven females and five males. Unilateral absence occurred in 8.6% of cases, affecting 18 females and eight males, with left and right frontal sinus absence variations among the latter.

Mean comparison of frontal sinus height between males and females

**Figure 3:**
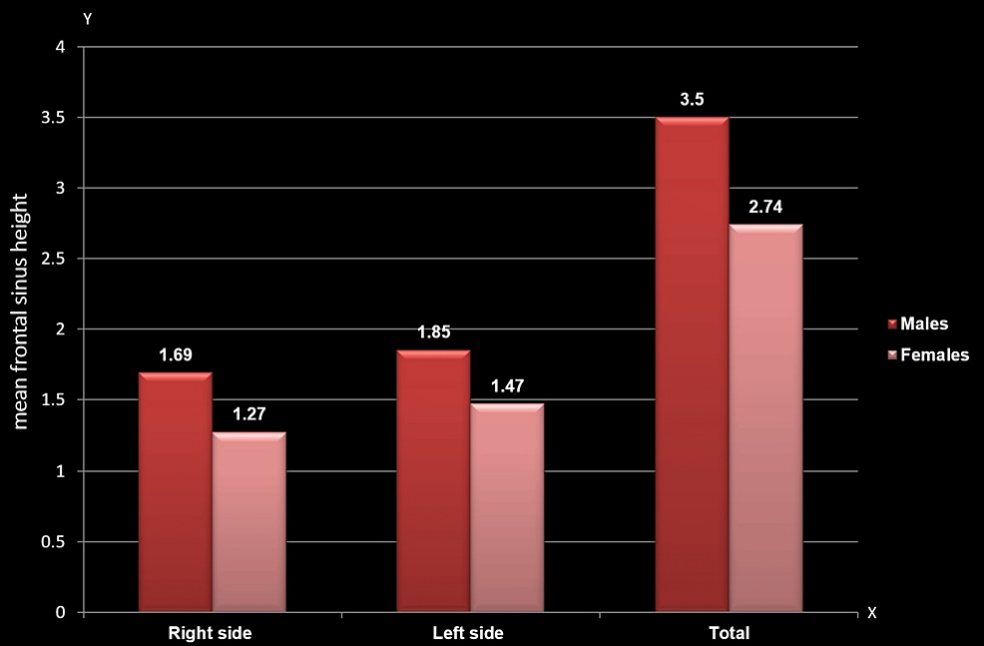
Mean comparison of frontal sinus height between males and females The y-axis represents the mean height of the frontal sinus, while the x-axis denotes the sides of the frontal sinus

A comparison of the mean frontal sinus height demonstrated a significant gender difference regardless of the side. The mean height scores were notably higher among males on the right side (1.69±0.72; p=0.000), left side (1.85±0.78; p=0.000), and overall (3.55±1.38; p=0.000) compared to females (1.27±0.54, 1.47±0.57, and 2.74±0.96, respectively) (Table 1, Figure 3).

**Table 1:** Mean comparison of frontal sinus height between males and females The Mann-Whitney U test was employed; p≤0.05 considered statistically significant The asterisk (*) indicates statistically significant findings

Frontal height	Right side		Left side		Total	
	Mean	SD	Mean	SD	Mean	SD
Males	1.6992	0.72363	1.8561	0.78319	3.5553	1.38598
Females	1.2778	0.54104	1.4710	0.57560	2.7488	0.96133
Mann-Whitney U value	5490.5		6077.5		5633.5	
P-value	0.000*		0.000*		0.000*	

Mean comparison of frontal sinus width between males and females

**Figure 4:**
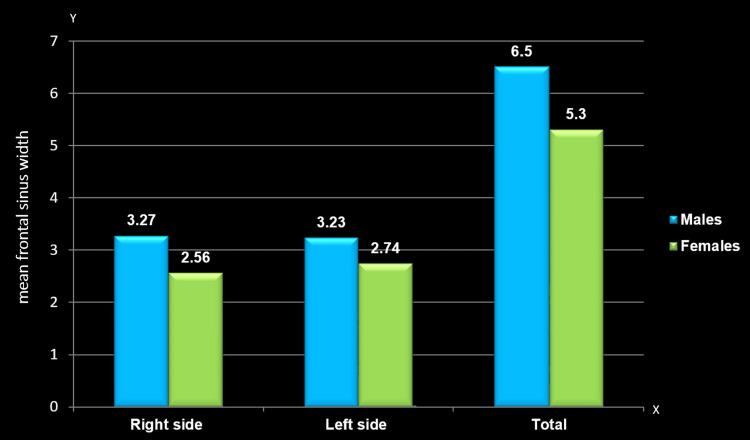
Mean comparison of frontal sinus width between males and females The y-axis represents the mean width of the frontal sinus, while the x-axis indicates the sides of the frontal sinus

A comparison of the mean frontal sinus width demonstrated a significant gender difference regardless of the side. The mean width scores were notably higher among males on the right side (3.27±3.48; p=0.000), left side (3.23±2.01; p=0.000), and overall (6.51±4.00; p=0.000) compared to females (2.56±1.11, 2.74±0.73, and 5.3±1.41, respectively) (Table 2, Figure 4).

**Table 2:** Mean comparison of frontal sinus width between males and females The Mann-Whitney U test was employed; p≤0.05 considered statistically significant The asterisk (*) indicates statistically significant findings

Frontal width	Right side		Left side		Total	
	Mean	SD	Mean	SD	Mean	SD
Males	3.2766	3.48191	3.2355	2.01754	6.5120	4.00856
Females	2.5658	1.11220	2.7425	0.73055	5.3083	1.41599
Mann-Whitney U value	6304.5		6122.5		5756.5	
P-value	0.000*		0.000*		0.000*	

Mean comparison of frontal sinus area between males and females

**Figure 5:**
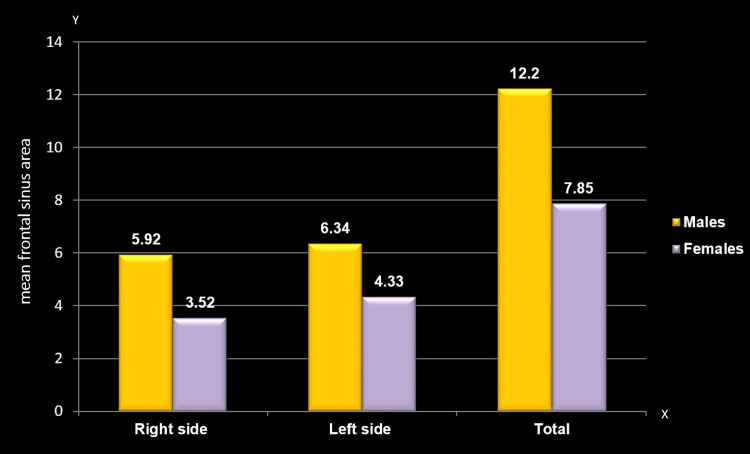
Mean comparison of frontal sinus area between males and females The y-axis represents the mean area of the frontal sinus, while the x-axis indicates the sides of the frontal sinus

A comparison of the mean frontal sinus area revealed a significant gender difference regardless of the side. The mean frontal sinus area scores were notably higher among males on the right side (5.92±6.54; p=0.000), left side (6.34±3.89; p=0.000), and overall (12.2±8.44; p=0.000) compared to females (3.52±2.24, 4.33±2.57, and 7.85±3.98, respectively) (Table 3, Figure 5).

**Table 3:** Mean comparison of frontal sinus area between males and females The Mann-Whitney U test was employed; p≤0.05 considered statistically significant The asterisk (*) indicates statistically significant findings

Frontal area	Right side		Left side		Total	
	Mean	SD	Mean	SD	Mean	SD
Males	5.9290	6.54160	6.3409	3.89195	12.2699	8.44035
Females	3.5247	2.24328	4.3314	2.57854	7.8561	3.98704
Mann-Whitney U value	5643.5		5842.5		5404	
P-value	0.000*		0.000*		0.000*	

Mean comparison of the number of partial septa between males and females

**Figure 6:**
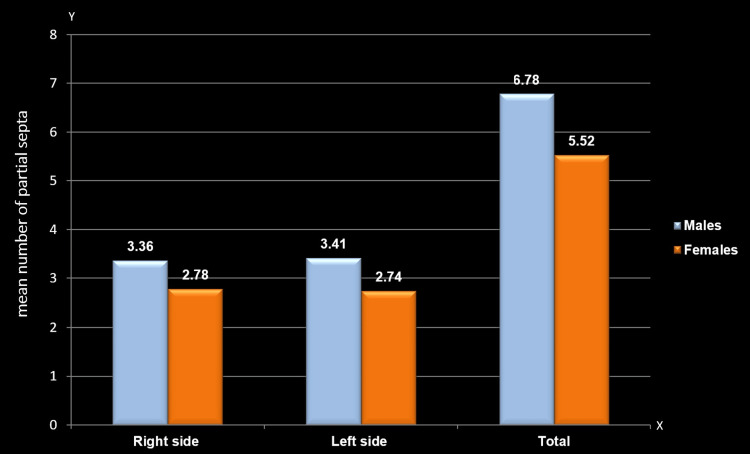
Mean comparison of partial septa between males and females The y-axis represents the mean number of partial septa in the frontal sinus, while the x-axis indicates the sides (right and left) of the frontal sinus

A comparison of the mean number of partial septa in the frontal sinus revealed a significant gender difference regardless of the side. The mean number of partial septa was notably higher among males on the right side (3.36±1.97; p=0.023), left side (3.41±1.88; p=0.002), and overall (6.78±3.26; p=0.001) compared to females (2.78±1.5, 2.74±1.37, and 5.52±2.41, respectively) (Table 4, Figure 6).

**Table 4:** Mean comparison of the number of partial septa between males and females The Mann-Whitney U test was employed; p≤0.05 considered statistically significant The asterisk (*) indicates statistically significant findings

Partial septa	Right side		Left side		Total	
	Mean	SD	Mean	SD	Mean	SD
Males	3.3650	1.97363	3.4161	1.88119	6.7810	3.26685
Females	2.7840	1.50045	2.7440	1.37315	5.5280	2.41493
Mann-Whitney U value	7191.000		6742.500		6627.000	
P-value	0.023*		0.002*		0.001*	

Distribution based on the outline of the upper border of the frontal sinus between males and females

**Figure 7:**
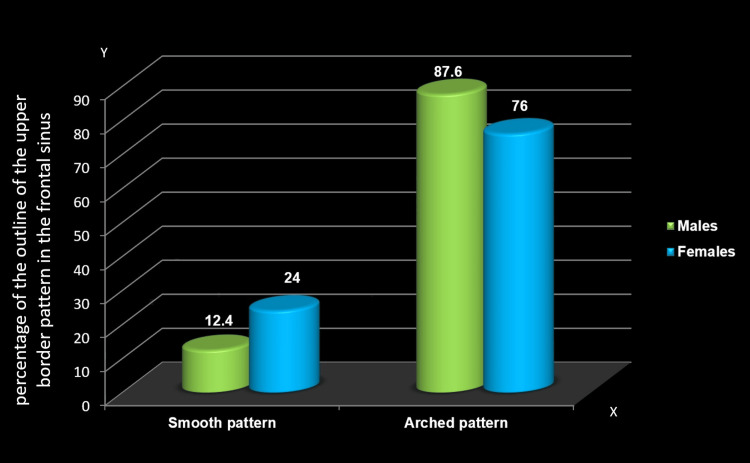
Distribution based on the outline of the upper border: right side The y-axis represents the percentage of the outline of the upper border pattern in the frontal sinus, while the x-axis indicates the different patterns of the upper border in the frontal sinus

**Figure 8:**
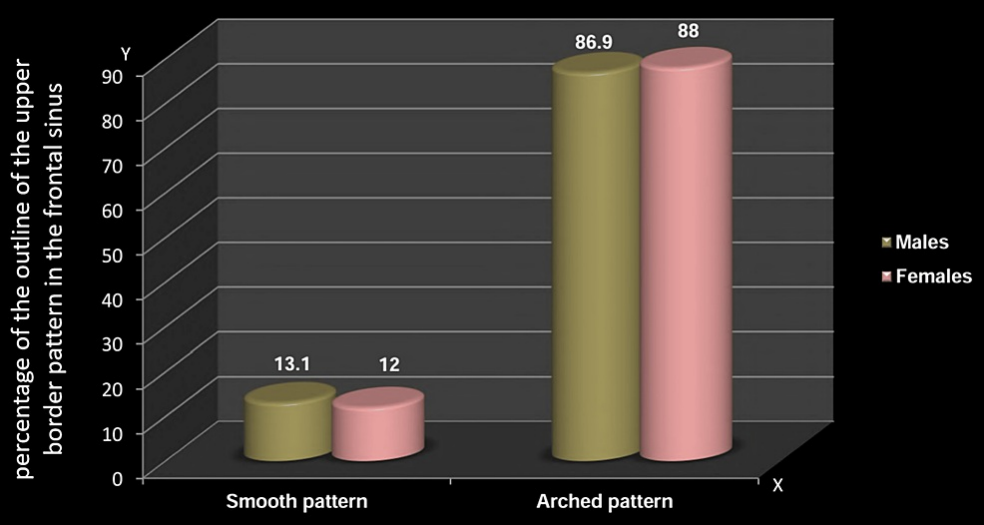
Distribution based on the outline of the upper border: left side The y-axis indicates the percentage of the outline of the upper border pattern in the frontal sinus, while the x-axis indicates the different patterns of the upper border in the frontal sinus

Distribution based on the outline of the upper border of the frontal sinus revealed a significant gender difference. On the right side, a significantly higher percentage of males and females exhibited an arched pattern (87.6% and 76%, respectively) compared to a smooth pattern (12.4% and 24%) (Table 5, Figure 7). However, on the left side, while the majority of males and females displayed an arched pattern (86.9% and 88%, respectively) in comparison to a smooth pattern (13.1% and 12%), no significant difference was observed (p=0.781) (Table 6, Figure 8).

**Table 5:** Distribution based on the outline of the upper border: right side The chi-squared test was employed; p≤0.05 considered statistically significant The asterisk (*) indicates statistically significant findings

Outline: right	Right side			
	Smooth		Arched	
	N	%	N	%
Males	17	12.4	120	87.6
Females	30	24	95	76
Chi-squared value	5.966			
P-value	0.011*			

**Table 6:** Distribution based on the outline of the upper border: left side The chi-squared test was employed; p≤0.05 considered statistically significant

Outline: left	Left side			
	Smooth		Arched	
	N	%	N	%
Males	18	13.1	119	86.9
Females	15	12	110	88
Chi-squared value	0.077			
P-value	0.781			

Distribution and comparison based on the symmetry of sinus between males and females

**Figure 9:**
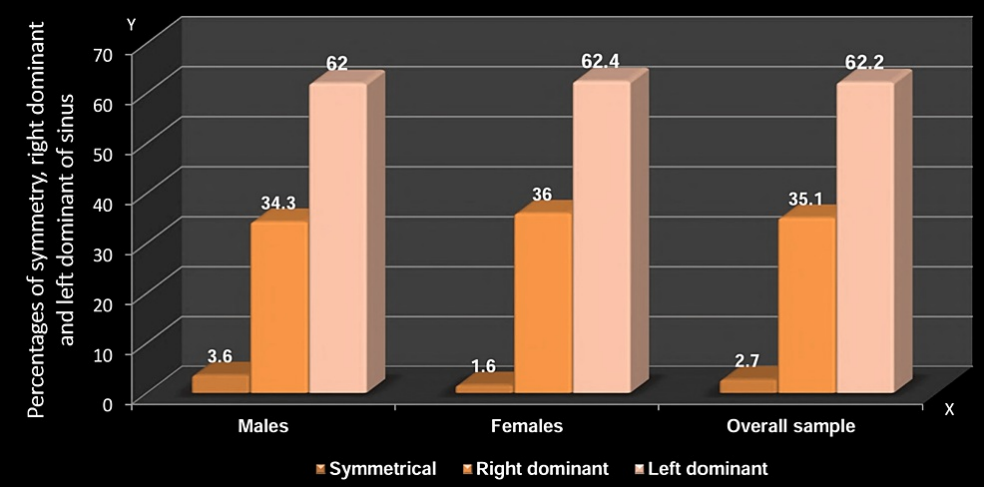
Distribution and comparison based on the symmetry of sinus between females and males The y-axis indicates the percentage of symmetry, right dominant, and left dominant of the frontal sinus, while the x-axis indicates males, females, and the overall sample

The distribution and comparison of sinus symmetry revealed a low symmetrical distribution of frontal sinuses in the study sample (2.7%). The majority exhibited dominance toward the left side (62.2%). No significant gender-based difference was observed in sinus symmetry, as both males (62%) and females (62.4%) predominantly displayed a left-dominant sinus configuration (p=0.582) (Table 7, Figure 9).

**Table 7:** Distribution and comparison based on the symmetry of sinus between females and males The chi-squared test was employed; p≤0.05 considered statistically significant

	Males		Females		Overall sample	
	Number	%	Number	%	Number	%
Symmetrical	5	3.6	2	1.6	7	2.7
Right dominant	47	34.3	45	36	92	35.1
Left dominant	85	62	78	62.4	163	62.2
Chi-squared value	1.082				-	
P-value	0.582				-	

Discriminant function analysis for sex determination based on frontal sinus characteristics

Utilizing discriminant function coefficients, an equation was formulated for sex determination based on frontal sinus characteristics, including height, width, area, and the number of partial septa. The derived formula from discriminant functional analysis is expressed as follows: gender=−2.410+0.422(frontal height)+0.002(frontal width)+0.069(area)+0.058(partial septa).

Upon applying this equation to the data, sex determination can be achieved using canonical centroids ranging between 0.344 and 0.377. Specifically, if the calculated product is close to 0.344, the proposed gender is male, while proximity to 0.377 suggests the proposed gender is female (Table 8, Table 9).

**Table 8:** Box's M statistics The asterisk (*) indicates statistically significant findings

Parameters	Male		Female		Wilks' λ	F ratio value	P-value
	Mean	SD	Mean	SD			
Height	3.5553	1.38598	2.7488	0.96133	0.898	29.407	0.000*
Width	6.5120	4.00856	5.3083	1.41599	0.963	10.117	0.002*
Area	12.2699	8.44035	7.8561	3.98704	0.902	28.395	0.000*
Partial septa	6.7810	3.26685	5.5280	2.41493	0.955	12.270	0.001*

**Table 9:** Canonical discriminate functions coefficient Sectioning points: males=0.344; females=0.377

Parameters	Discriminant function coefficient values
Height	0.422
Width	0.002
Area	0.069
Partial septa	0.058
Constant	-2.412

After applying the derived discriminant equation to the study sample for sex identification using frontal sinus characteristics, the accuracy achieved was 65.3%. Notably, there was a higher confidence in correctly diagnosing females (71.2%) compared to males (59.9%) (Table 9). Specifically, 59.9% of males were correctly predicted, while 71.2% of females were accurately identified. The overall accuracy for determining sex from the frontal sinus pattern was 65.3% (Table 10).

**Table 10:** Gender classification prediction and accuracy

		Gender	Predicted group membership		Total
			Male	Female	
Original	Count	Male	82	55	137
		Female	36	89	125
	%	Male	59.9	40.1	100.0
		Female	28.8	71.2	100.0

## Discussion

Various authors have explored the utility of different skeletal components for sexual dimorphism, focusing on paranasal sinuses, especially the maxillary and frontal sinuses, in gender determination. Frontal sinus radiographs have a longstanding history in forensics identifying unknown human remains. These paired cavities, situated behind the superciliary arches in the frontal bone, become radiographically visible at age 5, reaching maximum size by 20. Frontal sinus anatomy remains stable throughout life, undergoing gradual pneumatization in old age due to atrophic change. The frontal sinus is believed to serve functions such as regulating the respiratory system, relieving body pressure, reducing cranium weight, and assisting in thermal regulation [5]. Trauma, surgery, and pathology are recognized as factors modifying the frontal sinus, emphasizing its significance in forensic contexts [8].

Yoshino et al. [7] conducted a study using anteroposterior skull radiographs to identify Japanese individuals based on their frontal sinus patterns. Yoshino's classification includes parameters such as frontal sinus size, bilateral asymmetry, side superiority, outline of the upper border (left Ou1, right Ou2), partial septa (Ps), and supraorbital cells (Sc). The complexity of the facial skeleton has led to the development of various radiographic techniques. Two- and three-dimensional imaging, including radiographs, computed tomography (CT) scans, and cone-beam CT (CBCT) scans, has successfully aimed to establish the uniqueness of frontal sinus patterns [9]. However, conventional CT and CBCT have drawbacks, such as higher radiation doses compared to two-dimensional imaging and increased costs compared to radiographs [1].

The frontal sinus asymmetry has led to various attempts to identify individuals by analyzing sinus measurements from plain X-ray films. The skull's PA view, a non-angled radiograph, offers a comprehensive overview of the entire skull. This view aids in evaluating the upper, middle, and lower face, including the calvaria, sinuses (frontal, ethmoidal, and maxillary), alveolar bone, and teeth. The commonly used PA view, also known as the Caldwell view, is particularly effective for assessing frontal and ethmoidal sinus morphology. PA skull radiographs are also utilized for legal purposes. PA is preferred over Water's view due to image foreshortening, and the advantage of a two-dimensional image lies in its easy production and widespread availability [9]. The prevalent statistical method for sex determination is discriminant analysis, primarily employed to categorize individuals into two or more groups based on a set of measurements. DFA facilitates the assignment of a subject with an unknown identity to one of the specified groups through linear multivariate observation [10].

The present study examines 300 digital PA cephalograph radiographs of 150 males and 150 females aged 18-30 years. The digital radiograph assessment includes the analysis of Yoshino's frontal sinus pattern, covering parameters such as frontal sinus size, bilateral asymmetry, side superiority, upper border outline, partial septa, and supraorbital cells.

The study includes a sample aged between 18 and 30 years, with a mean age of 24 years, aligning with the research conducted by Verma et al. [11], Camargo et al. [8], Gadekar et al. [12], Denny et al. [13], and Hussain et al. [14]. However, Uthman et al. [15] included individuals aged 20-49 years, while Verma et al. [16] and Khan et al. [17] restricted their samples to those aged between 20 and 45 years. Goyal et al. [4], Kiran et al. [18], and Pandeshwar et al. [9] conducted studies involving individuals aged between 21 and 54 years. Studies by Patil et al. [6], Hamed et al. [19], Selarka et al. [20], and Eboh et al. [21] reported slightly higher mean ages compared to the present study. Cossellu et al. [22], Soman et al. [23], and Shireen et al. [1] had a lower age limit lower than the present study. The decision to limit the sample to young adults is rationalized by the fact that frontal sinuses complete their development by approximately 20 years and remain stable, with observable changes in old age, including thinning walls and an apparent increase in size [12].

In this study, we analyzed 300 randomly selected digital PA radiographs to evaluate the frontal sinus, aligning with similar studies conducted by Belaldavar et al. [5] on 300 Indian-origin radiographs and Khan et al. [17] on 300 radiographs. Additionally, Verma et al. [11] and Soman et al. [23] investigated 80 and 200 digital PA radiographs, respectively. Goyal et al. [4] focused on 100 digital paranasal sinus (PNS) radiographs. Modifications of the PNS view, the Caldwell view, were explored by Mathur et al. [24] and Kaur et al. [25] on 40 and 50 radiographs, respectively. Camargo et al. [7] conducted a similar study on 100 radiographs. Uthman et al. [15] employed CT scans on 90 patients, while Tatlisumak et al. [26] used 100 CT scans. Boyacioglu et al. [27] studied frontal sinuses using CBCT on 97 scans, and Denny et al. [13] conducted a similar study using 100 CBCT images. While conventional CT and CBCT have higher doses and cost drawbacks, this study employs a simple and cost-effective methodology using digital PA radiography, ensuring high accuracy in human identification through frontal sinus analysis [1].

In the 300 radiographs studied, bilateral aplasia was observed in 12 cases (4%), with seven females (2.3%) and five males (1.6%). Unilateral absence was seen in 8.6% of cases involving 18 females (6%) and eight males (2.6%). Of the males with unilateral absence, four showed left frontal sinus absence, and four showed right frontal sinus absence. Among the females, 13 exhibited an absence of the right frontal sinus, and five had an absence of the left frontal sinus. This study aligns with findings from Belaldavar et al. [5] and Mathur et al. [24], both reporting frontal sinus absence in 4% of cases. Bilateral absence rates vary in different studies, ranging from 5.50% [28] to 8% [12,17]. Bilateral aplasia was reported as 2% in studies by Chaudhary and Singh [29] and Goyal et al. [4]. Unilateral aplasia rates also differ, with studies by Chaudhary and Singh [29], Pandeshwar et al. [9], and Kanjani et al. [30] reporting rates of 6%, 8%, and 12.2%, respectively. Interestingly, this study notes a higher incidence of frontal sinus absence in females compared to males, consistent with findings in Turkish, Japanese, and Alaskan Eskimo populations [31].

In the present study, all measured variables (height, width, and area) consistently exhibited larger mean values in males compared to females, likely influenced by genetic factors rather than nutritional, hormonal, or muscular factors. Specifically, significant differences were found in males' right and left frontal sinus height (1.69±0.72 cm, 1.85±0.78 cm), width (3.27±3.48, 3.23±2.01), and area (5.92±6.54 cm², 6.34±3.89 cm²) compared to females. Comparison with other studies revealed variations in findings. Belaldavar et al. [5] and Camargo et al. [8] reported lower measurements for frontal sinus dimensions. Soman et al. [23] and Kaur et al. [25] found smaller frontal sinus length and area values. Nethan et al. [28] and this study showed no statistically significant differences in frontal sinus dimensions between males and females. In conclusion, the current study suggests that left height and left area are better predictors for sex determination based on frontal sinus measurements. The observed measurements in this study generally exceed those reported in comparable studies, indicating potential population or methodological variations.

In the present study, the left frontal sinus was larger than the right in both males and females, aligning with findings from studies by Camargo et al. [8], Pondé et al. [31], and Raoof et al. [32]. This contrasts with results from Belaldavar et al. [5], Hussain et al. [14], and Gadekar et al. [12]. This asymmetry is attributed to the independent development of frontal sinuses, with one side commonly larger than the other. The morphology of frontal sinuses in both genders exhibited asymmetry and distinct differences.

In this study, males exhibited a statistically significant and higher mean of partial septa compared to females, aligning with the findings of Pandeshwar et al. [9]. There was a highly significant difference in the presence of right partial septa, consistent with Khan et al.'s study [17]. However, studies by Yoshino et al. [7], Soman et al. [23], Aydinlioglu et al. [33], Tang et al. [34], and Gadekar et al. [12] did not find statistical significance in partial septa.

The current study noted significant gender-based differences in the frontal sinus upper border outline, favoring both males' and females' scalloped patterns on the right side. This aligns with Gadekar et al.'s findings [12] and is consistent with the Chinese Han population but contrasts with the Italian population [34]. Pandeshwar et al. [9] reported left scallop differences, while Soman et al. [23] found no statistical distinction in the upper border outline. Population ethnicity may contribute to variations in partial septa and scalloped upper border patterns.

In the present study, frontal sinus symmetry was low (2.7%), predominantly left-sided (62.2%), with no significant gender-based differences. This differs from Shireen et al. [1] (83.20% symmetry). Left-dominant asymmetry was 6.98%, and right-dominant asymmetry was 9.82%. Chaudhary and Singh [29] found 62% symmetry, while Tang et al. [34] reported 43.1% in the Japanese population. Frontal sinus asymmetry is common on both sides due to unequal dipole resorption during development [6]. In the current study, side superiority and supraorbital cells lacked significance, consistent with Soman et al. [23], Aydinlioglu et al. [33], and Gadekar et al. [12]. This differs from the findings of Pandeshwar et al. [9]. Tatlisumak et al. [26] didn't consider supraorbital cells, and their system eliminated 93% of formulas, rising to 98% with added measurements.

The present study utilized discriminant analysis for sex determination based on frontal sinus patterns, establishing a cut-off score. The accuracy in sex determination was 65.3%, with higher confidence in females (71.2%) than males (59.9%). This aligns with various studies, including Pandeshwar et al. [9] and Belaldavar et al. [5], showing comparable or higher accuracy. However, studies by Goyal et al. [4] reported lower accuracy. The current study aligns with Pandeshwar et al. [9], using Yoshino's method, and shows 65.7% accuracy in sex determination. Similar results were found in studies by Belaldavar et al. [5] and Kiran et al. [18]. Other studies, like Camargo et al. [8] and Uthman et al. [15], reported higher accuracy. Tatlisumak et al. [26] achieved 98% success using the FSS system. Notably, the study emphasizes the reliability of frontal sinus patterns for sex determination, aligning with other studies with varying accuracy rates.

Limitations 

While the study provides valuable insights into gender determination using frontal sinus morphometrics, it is essential to acknowledge a few limitations within the study. Firstly, the study's sample is limited to individuals aged 18-30 years, which may not represent the entire population accurately. Additionally, the study focuses on a specific ethnic group, potentially limiting the generalizability of the findings to other populations. Furthermore, the final sample size after exclusions is relatively small. A larger sample size would increase the study's statistical power and reliability of the results. Finally, the study does not consider potential external factors that could influence frontal sinus morphology, such as hormonal fluctuations, nutritional status, or environmental factors. These factors may contribute to variability in frontal sinus characteristics and should be considered in future research.

## Conclusions

This study validates the reliability and potential of frontal sinus morphometrics for gender identification, utilizing Yoshino's classification. It complements established methods such as pelvic and skull analysis. The results reveal apparent gender-based differences in frontal sinus dimensions, with males consistently exhibiting more extensive measurements compared to females. Furthermore, asymmetry in frontal sinus morphology and variations in the presence of partial septa and upper border outlines are noted, highlighting the uniqueness of individual sinus patterns. These findings underscore the importance of considering population-specific variations in forensic analyses. Utilizing discriminant function analysis, the study establishes a reliable methodology for sex determination based on frontal sinus characteristics, achieving an overall accuracy of 65.3%. While the accuracy rates vary slightly between genders, the study demonstrates a higher confidence in correctly identifying females compared to males. Nevertheless, the results affirm the reliability of frontal sinus patterns as a supplementary tool for gender identification in forensic investigations. This research advances our understanding of frontal sinus morphology and its utility in forensic anthropology, emphasizing the importance of standardized methodologies and population-specific analyses in enhancing accuracy and reliability in forensic identification processes. These findings have implications for forensic practitioners, providing a valuable resource for victim identification, criminal profiling, and legal proceedings.
